# Evidence of the Effectiveness of Primary Brachial Plexus Surgery in Infants With Obstetric Brachial Plexus Palsy–Revisited

**DOI:** 10.1177/2329048X17709395

**Published:** 2017-05-25

**Authors:** Amel A. F. El-Sayed

**Affiliations:** 1 Department of Obstetrics and Gynecology, King Saud University, Riyadh, Saudi Arabia

**Keywords:** Erb’s palsy, brachial plexus, primary surgery, conservative management

## Abstract

A recent systematic review questioned the effectiveness of primary surgery in infants with obstetric brachial plexus palsy. At our center, the indication for primary surgery in infants with upper Erb’s obstetric palsy is the lack of active elbow flexion at age 4 months. The current study compares the outcome of motor recovery in 2 groups of infants with upper Erb’s palsy: one group (n = 9) treated surgically between age 4 and 5 months, and another group (n = 9) treated conservatively despite the lack of active elbow flexion at age 4 months. The only reason for not doing the surgery in the latter group was refusal by the parents. The scores of motor recovery were collected at the 2-year follow-up visit, and they were significantly better in the surgical group. The study demonstrates the effectiveness of primary surgery in infants with upper Erb’s obstetric palsy compared to conservative management.

Obstetric brachial plexus surgeons are the decision-makers regarding the indications for primary brachial plexus reconstruction (nerve grafting or nerve transfers) in infants with obstetric brachial plexus palsy. These surgeons strongly believe that the lack of spontaneous motor recovery of the affected roots of the brachial plexus in the age of first few months is a clear indication for surgery.^1^ Gilbert^2^ and others^3^ recommend surgery at age 2 to 3 months for infants with total palsy and concurrent Horner syndrome. This recommendation is based on the fact that Horner syndrome is usually an indicative of severe lower root injury or T_1_ avulsion.^3^ The timing and indications of primary surgery for Erb’s obstetric palsy vary according to the criteria put by the obstetric brachial plexus surgical centers. Gilbert’s^2^ center recommends surgery at age 3 months if there is no active elbow flexion at the 3-month visit. Al-Qattan’s^4^ center recommends surgery at age 4 months if there is no active elbow flexion against gravity at the 4-month visit. Clarke’s^1^ center puts a scoring system for multiple joint movements during the age of first 9 months, and surgery would be indicated if a satisfactory score is not reached. Despite the lack of comparative studies, the surgeons consider conservative management unacceptable in infants with inadequate spontaneous recovery in the age of first few months.^1-4^

The effectiveness of primary brachial plexus surgery for infants with obstetric brachial plexus palsy is a controversial issue in the nonsurgical and pediatric literature. Socolovsky et al^5^ reviewed the literature and concluded that there is considerable published evidence to favor primary nerve surgery as the initial therapeutic step in infants who do not recover spontaneously. In contrast, the systematic review of Bialocerkowski and Gelding^6^ was not able to determine whether primary surgery is effective in increasing the functional recovery. The authors^6^ concluded that comparison groups are required to determine the relative effectiveness of surgery compared with conservative management.

The indication for primary surgery in Erb’s obstetric palsy in our center is lack of active elbow flexion against gravity by age 4 months. The aim of the current study is to compare the outcome of motor recovery in 2 groups of infants with upper Erb’s palsy (ie, involving the C_5_ and C_6_ roots): one group treated surgically by primary brachial plexus reconstruction between age 4 and 5 months, and another group treated conservatively despite the lack of active elbow flexion against gravity at age 4 months. The only reason for not doing the primary surgery in the latter groups was refusal by the parents.

## Patients and Methods

A retrospective chart review of 902 infants with obstetric brachial plexus palsy treated at our center between 1995 and 2016 revealed 9 cases with upper Erb’s palsy (C5–C6 palsy) who were offered primary brachial plexus surgery at the age of 4 months (because of lack of active elbow flexion against gravity), with refusal of the parents. All 9 cases did not have any primary surgery and had a minimum follow-up of 2 years after the decision for surgery made at age 4 months. These 9 cases were included in the current study as the “conservative” group or group 1. These cases were the only 9 (of the 902 infants) who had upper Erb’s palsy and who were offered primary brachial plexus surgery at age 4 months (because of lack of active elbow flexion against gravity), with refusal of the parents. Another 9 randomly selected infants with upper Erb’s palsy who underwent primary brachial plexus surgery between 4 and 5 months (because of lack of active elbow flexion against gravity at age 4 months) were included in another “surgical” group or group 2. The data of all patients (n = 107) with upper Erb’s palsy and who underwent primary brachial plexus surgery between 4 and 5 months were identified, and the cases were given serial numbers. Nine cases were randomly selected by the computer by simple random sampling.

The following data were collected: diabetes mellitus in the mother, the presentation of the baby, method of delivery, history of shoulder dystocia, and the presence of concurrent injuries (such as phrenic nerve palsy, asphyxia, skull injury, and clavicular/long bone fractures). The authors do not routinely do nerve conduction, electromyography (EMG), or magnetic resonance imaging (MRI) in infants with obstetric brachial plexus palsy; hence, such data were not available for collection. In the surgical group, data regarding timing of surgery, intraoperative findings, and the method of brachial plexus reconstruction were also collected.

At our center, motor assessment of the affected limb is documented at every visit for all infants with obstetric brachial plexus palsy, and the authors have criteria for what they consider as a “satisfactory function.” In upper Erb’s palsy, relevant functions are shoulder abduction and external rotation (the motor function of the C5 root) and elbow flexion (the motor function of the C6 root). Table 1 shows the assessment methods as well as the definitions of satisfactory functions used in the current study. In the conservative group (group 1), data of the motor assessment were collected at age 4 months and at the first visit after the age 2 years and 4 months (ie, at 2 years after the decision for surgery was made but refused by the parents). In the surgical group (group 2), the same data were collected at age 4 months and at the first visit after the age 2 years and 4 months. (ie, at 2 years after surgery). The percentages of satisfactory functions at final assessment were compared between the 2 groups using the Fisher exact test. A *P* value less than .05 was considered significant.

**Table 1.** Motor Assessment in Children With Upper Erb’s Palsy.

Function	Scoring or Measurement of Function	Definition of a Satisfactory Functional Outcome
Shoulder abduction	Measured as degrees of shoulder abduction	Abduction of 120° or more
Shoulder external rotation	1 = The hand reaches the abdomen or thorax, 2 = the hand reaches the mouth, 3 = the hand reaches the ear, 4 = the hand reaches the occiput, and 5 = normal external rotation	A score of 3 or more
Elbow flexion	0 = No motion, 1 = active motion with gravity eliminated, 2 = active motion against gravity, 3 = active motion against resistance reaching <1/2 normal range, 4 = active motion against resistance reaching >1/2 normal range, and 5 = normal	A score of 4 or 5

## Results

Both groups had equal number of patients (n = 9). Four mothers in each group have gestational diabetes. The presentation of the baby was cephalic in all 18 babies, and all were delivered vaginally at term. A history of “difficult” delivery or shoulder dystocia was documented in every case. One patient in each group had a concurrent ipsilateral clavicular fracture. One patient in the second group had a small subgaleal hematoma. None of the infants had long bone fractures, skull fractures, phrenic nerve injury, or asphyxia requiring admission to the Neonatal Intensive Care Unit (NICU).

Timing of surgery in the surgical group ranged from age 4 months to 4 months and 3 weeks. The reasons for the slight delay once the decision for surgery was made were either the unavailability of operative time or mild unrelated sickness (diarrhea or upper respiratory tract viral infection). The intraoperative findings in surgical group (n = 9) were ruptures of both C5 and C6 roots in 7 cases; in the remaining 2 cases (case numbers 2 and 4 shown in Tables 2-4), there was a rupture of the C5 root and avulsion of the C6 root. The method of brachial plexus reconstruction in the former 7 cases with double root rupture was intraplexus neurotization using sural nerve grafts coapted between the C5/C6 roots proximally and the upper trunk/suprascapular nerve distally. In the latter 2 cases, intraplexus neurotization was used to reconstruct the posterior division of the upper trunk and suprascapular nerve (for shoulder abduction/external rotation reconstruction). Since the C6 root was avulsed in the latter 2 cases, biceps reinnervation in these 2 cases was done using a distal nerve transfer: a fascicle from the ulnar nerve was transferred to the biceps nerve in the arm in one case, and a fascicle from the median nerve was transferred to the biceps nerve in the arm in the other case, using the techniques described in the surgical literature.^7,8^

**Table 2.** Shoulder Abduction in the 2 Study Groups at Age 4 Months and at Final Assessment 2 Years Later.

Case No.	Shoulder Abduction in Degrees in the Conservative Group 1 (n = 9)		Shoulder Abduction in Degrees in the Surgical Group 2 (n = 9)	
	Abduction at Age 4 Months	Abduction at Final Assessment	Abduction at Age 4 Months	Abduction at Final Assessment
1	30°	40°	20°	160°
2	20°	40°	30°	140°
3	30°	30°	30°	170°
4	10°	30°	10°	60°
5	20°	30°	20°	70°
6	30°	50°	20°	180°
7	10°	30°	20°	130°
8	20°	30°	10°	150°
9	20°	30°	30°	90°

**Table 3.** Shoulder External Rotation Score^a^ in the 2 Study Groups at Age 4 Months and at Final Assessment 2 Years Later.

Case No.	Shoulder External Rotation Score^a^ in the Conservative Group 1 (n = 9)		Shoulder External Rotation Score^a^ in the Surgical Group 2 (n = 9)	
	External Rotation at Age 4 Months	External Rotation at Final Assessment	External Rotation at Age 4 Months	External Rotation at Final Assessment
1	1	2	1	4
2	1	2	1	3
3	1	1	1	4
4	1	1	1	2
5	1	1	1	2
6	1	2	1	4
7	1	1	1	4
8	1	1	1	4
9	1	1	1	2

^a^Shoulder external rotation score is as per Table 1.

**Table 4.** Elbow Flexion Score^a^ in the 2 Study Groups at Age 4 Months and at Final Assessment 2 Years Later.

Case No.	Elbow Flexion Score^a^ in the Conservative Group 1 (n = 9)		Elbow Flexion Score^a^ in the Surgical Group 2 (n = 9)	
	Elbow Flexion at Age 4 Months	Elbow Flexion at Final Assessment	Elbow Flexion at Age 4 Months	Elbow Flexion at Final Assessment
1	0	0	1	4
2	0	0	1	5
3	1	4	0	4
4	0	1	0	5
5	1	1	0	5
6	0	0	0	5
7	0	1	0	4
8	0	3	0	5
9	0	3	0	5

^a^Elbow flexion score is as per Table 1.

The definition of a satisfactory shoulder abduction at our center is abduction of 120° or more. Table 2 shows the data of shoulder abduction in both groups. At age 4 months, all infants in both groups had a poor shoulder abduction ranging from 10° to 30°. In the conservative group, the abduction slightly improved in all infants after 2 years to reach a range of 30° to 50°, but none reached a satisfactory score (0% satisfactory score in group 1). In contrast, 6 of the 9 surgical cases qualified for a satisfactory outcome (66.7% satisfactory score in group 2). The difference between the 2 groups was significant (*P* = .005).

Table 3 shows the data for shoulder external rotation. At age 4 months, all infants in both groups had a score of 1 for external rotation. At the 2-year assessment, a satisfactory functional score (a score of 3-5) was not seen in any of group 1 patients (0%), but it was seen in 6 (66.7%) of the 9 patients in group 2. The difference between the 2 groups was significant (*P* = .005).

Table 4 shows the data for elbow flexion. At age 4 months, all infants in both groups had a score of 0 to 1 for elbow flexion. At the 2-year assessment, only 1 of the 9 (11.1%) patients in group 1 had a satisfactory score. In contrast, all children (100%) in group 2 qualified for a satisfactory score for elbow flexion. The difference between the 2 groups was highly significant (*P* < .0001).

## Discussion

Our study in the first investigation in the literature comparing conservative management to primary surgical reconstruction of the brachial plexus in obstetric palsy. The main reason for the lack of similar studies in the literature is probably the belief of surgeons that a trial of conservative therapy is not ethical. In our study, the conservative management group were infants who did not undergo surgery because of refusal of surgery. The authors documented this refusal in the file for medicolegal purposes. The study clearly demonstrated that conservative management is not acceptable for upper Erb’s obstetric palsy using the lack of active elbow flexion against gravity at age 4 months as the indication for primary brachial plexus surgery. Despite the small number of patients in each group, the differences in the outcome at all relevant motor functions reached statistical significance.

It is important to note that the authors do not do MRI for infants with obstetric brachial plexus palsy; hence, the nature/severity of root injury (ie, rupture vs avulsion) was unknown prior to surgery. The main reasons for not doing MRI are the need for general anesthesia for MRI in infants and the fact that the decision for primary surgery in Erb’s obstetric palsy at our center is based on 1 clinical criterion which is the lack of active elbow flexion against gravity by age 4 months (regardless of any other radiological or electrophysiological findings). The use of this single clinical criterion as the indication for surgery was previously reported by Al-Qattan^4^ who also found that all these infants were found to have either root avulsion or rupture upon surgical exploration.

Adult traumatic upper Erb’s palsy is usually caused by motor vehicle accidents or falls from heights. In these cases, the high force and sudden impact usually result in avulsion of both C5 and C6 roots. Clinically, the shoulder is flail and there is flaccid paralysis of the biceps. In adults, neurophysiological testing is reliable in diagnosing root avulsion. Primary surgical exploration in these cases is not a controversial issue; good results in adults were reported using multiple nerve transfers.^9^ In obstetric brachial plexus palsy, isolated C5/C6 injury results from gradual exertion of a low traction force. Hence, double root avulsion is extremely rare, and the injury is usually a stretch injury of the roots resulting in a “neuroma-in-continuity.” Axons still pass through the neuroma and hence the shoulder is not flail; instead, there is usually weak active shoulder abduction along with an internal rotation/abduction contracture. Furthermore, after a variable period of conservative management, most children will demonstrate active motion of the elbow, and in our series, 1 child even recovered a scored 4 elbow flexion (Table 4). What complicates matters is the fact that EMG and nerve conduction studies in infants with obstetric brachial plexus palsy are not as reliable as in adults. A recent review by Malessy et al^10^ concluded that the absence of “gold standard” for the electrophysiological assessment of the severity of the brachial plexus lesion in infants makes prognostic studies in obstetric brachial plexus palsy complex. Hence, the only way to investigate the effectiveness of primary surgery in obstetric brachial plexus palsy is to compare surgery to conservative management.

The results of surgical reconstruction of the brachial plexus in our series are similar to those reported in the literature.^11-13^ The surgical results for elbow flexion have been excellent in all series. In contrast, the surgical results for shoulder abduction and external rotation are known to be inferior to the results at the elbow because of other factors such as the development of osseous deforming around the shoulder and development of contractures of shoulder adductors/internal rotators.^14^ Furthermore, the postsurgical outcome in shoulder external rotation is also affected by the frequent occurrence of a second site of scarring or entrapment of the suprascapular nerve at the suprascapular notch.^15^

In conclusion, our study provides clear evidence supporting the effectiveness of primary surgery in infants with upper Erb’s obstetric palsy.
